# Segmentation and regional analysis: labor typologies and geographic inequality in Antofagasta and La Araucanía, Chile

**DOI:** 10.3389/fsoc.2025.1636282

**Published:** 2025-10-08

**Authors:** Osvaldo Blanco, Dasten Julián-Véjar, Alejandro Osorio-Rauld

**Affiliations:** ^1^Faculty of Health and Social Sciences, University of the Americas, Santiago, Chile; ^2^Global Dynamics of Social Policy, University of Bremen, Bremen, Germany; ^3^SWOP Institute, University of Witwatersrand, Johannesburg, South Africa; ^4^University of Alicante, Alicante, Spain

**Keywords:** labor segmentation, regional analysis, regional employment structure, Antofagasta and La Araucanía, labor precarity

## Abstract

This article examines the geographic variability of labour markets in the Chilean regions of Antofagasta and La Araucanía, emphasizing their structural similarities and differences. An inter- and intraregional perspective is applied to identify common and specific elements in both territories, drawing on multivariate sociodemographic, contractual, and occupational data. Through factorization and cluster analysis, typological systems of labour segmentation are constructed for each region, located at opposite ends of the country. The results reveal the heterogeneity of regional labour matrices, encompassing both regular and protected employment as well as unregulated, temporary, informal, and precarious arrangements. The study also considers the role of urban and rural contexts in shaping productive activities and labour conditions. While extractive industries represent a shared feature of Chile’s productive matrix, the findings show unequal segmentation systems across regions. These differences highlight persistent gender inequalities in access to decent and protected work, along with differentiated patterns of labour insertion for Indigenous peoples and migrants across urban and rural areas. The analysis contributes to understanding regional labour dynamics and underscores the need for territorially differentiated policies that acknowledge the structural diversity of employment conditions in northern and southern Chile.

## 1 Introduction

There are diverse forms of organization within capitalist systems. Not all adopt the same development models, welfare policies, institutional structures, or present the same productive configuration or diversification (Hall and Soskice, 2001; Dibben and Collins, 2012). In peripheral societies, it is crucial to highlight the specificities of their capitalist models in order to design strategies aimed at promoting population well-being. These strategies may range from promoting industrialization to implementing policies aimed at reducing inequality and informal employment. In general terms, the Latin American development model is distinguished by several cross-cutting factors, including a strong presence of foreign companies, a dependence on raw material exports, limited industrialization, and deep social inequalities (Alister et al., 2021).

However, it is possible to affirm that there are structural elements linked to the geographic variability of capitalism that reveal specific characteristics in each local labor market. In Chile, there is a marked internal productive heterogeneity, determined by the country’s geographic diversity and inequality. This reality gives rise to differentiated regional contexts that configure diverse productive landscapes, where different sectors and economic actors coexist, as well as specific territorial dynamics in the consolidation of enclaves and their articulation with the national and global economy. These processes have been shaped by the peripheral and dependent nature of the Chilean economy, configured through the country’s colonial legacy and state intervention in the promotion and prioritization of certain productive sectors over others. As a result, a geographically segmented labor market has been structured (Blanco, 2019), characterized by high levels of job insecurity and informality (Blanco and Julián, 2019).

In the north of the country, the Antofagasta region is connected to global trade circuits, especially around mining. Meanwhile, in the south, the Araucanía region is frequently portrayed as the poorest in the country. While Antofagasta is considered a dynamic region with a high GDP per capita, Araucanía is viewed as economically stagnant, backward, and with a low GDP per capita (ICHEM, 2024). In Antofagasta, mining is the largest contributor to regional GDP, although trade generates more employment. In Araucanía, although the agricultural, forestry, and livestock sectors are relevant to regional GDP, the most predominant sectors are construction, transportation, housing and real estate services, and personal services. However, no economic sector in Araucanía contributes as much to GDP as mining does in Antofagasta.

Both regions have key productive sectors in the country’s social and economic history: mining and agriculture. A key milestone was the occupation of La Araucanía (1860–1883), which resulted in the dispossession and distribution of land among the military, mestizos, and settlers, along with the creation of indigenous reserves (Bengoa, 2000). Currently, the region combines forestry and agricultural exports with sharecropping, family units, and peonage models. The Antofagasta region, especially the cities of Calama and Chuquicamata, has been central to the Chilean economy since the 19th century, following its occupation during the War of the Pacific (1879–1884), which involved the annexation of this territory and its indigenous population (Aymara, Likan, and Lama, Antau, Quechua Colla and Diaguitas). This area has evolved from a mining enclave towards processes of clustering and productive chaining (Curverwell, 2000), also generating urbanization processes and, especially in recent years, a migratory dynamic from neighboring countries.

**MAP 1:**
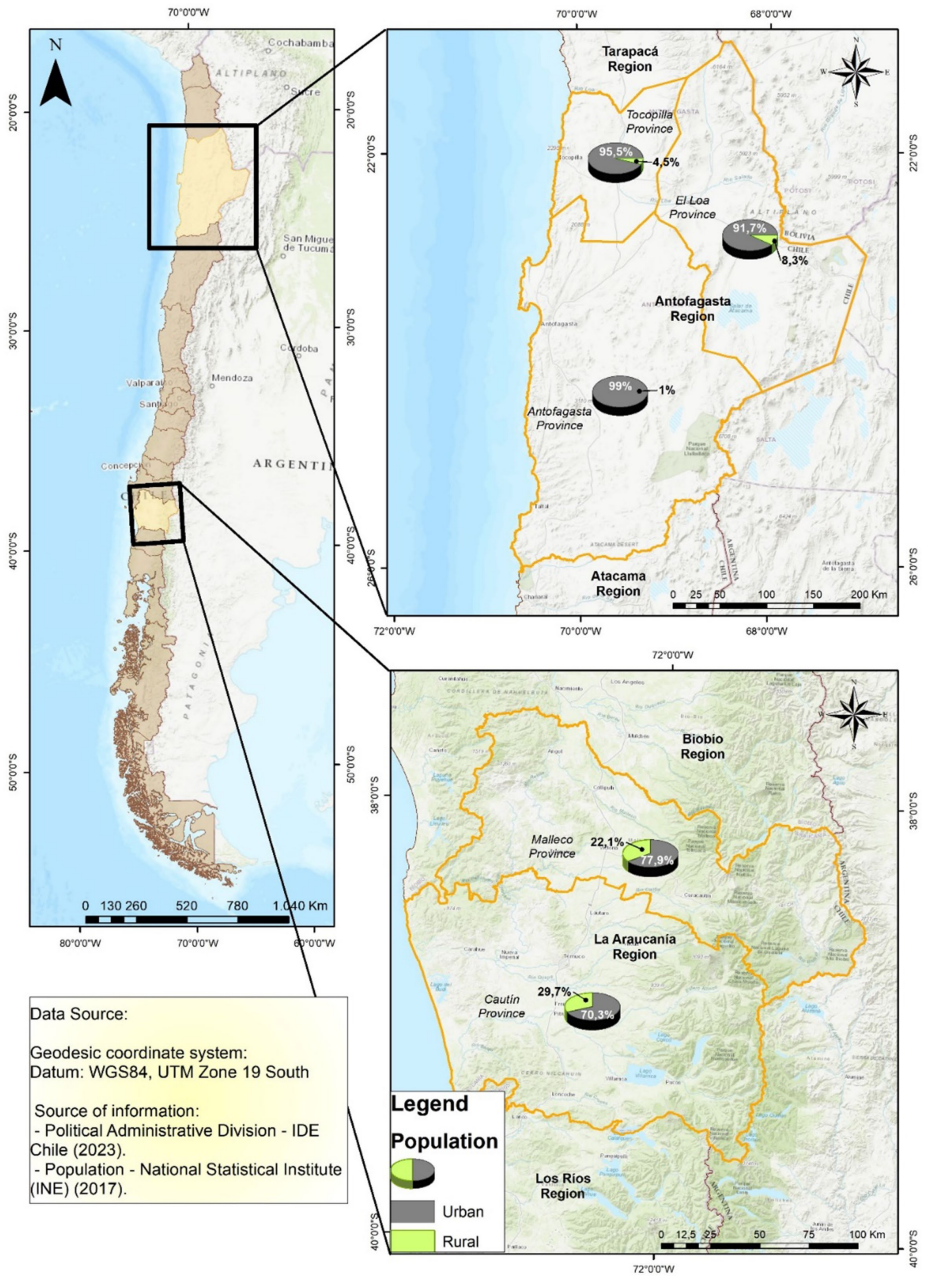
Distribution of the urban and rural population in the regions of Antofagasta and La Araucanía, Chile. Source: Prepared by the authors using CASEN 2022 data. Antofagasta (*N* = 321,811), La Araucanía (*N* = 405,225).

These processes have not been homogeneous, as reflected in the fact that Antofagasta’s economic growth has driven an intense urbanization process, relegating the rural world to a condition of isolation and a smaller relative territorial scale (Map 1). In contrast, La Araucanía has a significant presence of rural territories, as well as urban territories and population. However, the importance of urban dynamics in La Araucanía does not exhibit the strength and intensity observed in Antofagasta.

The historical development and the increasingly complex economic and productive physiognomy of both regions has led to a phenomenon known in the literature as labor market segmentation (Cruz Gómez et al., 2019; Bensusán Areous, 2022; López-Roldán et al., 2021). In particular, segmentation dynamics linked to the intersection of class, gender, and race operate (Vigoya, 2016; Blanco et al., 2022; Blanco and Álvarez, 2025), associated both with the specificity and geographic variability of productive matrices in peripheral-dependent capitalism and with the persistence and renewal of power relations (Julián-Vejar and Valdés Subercaseux, 2022). Within this framework, the analysis focuses on the specificity of labor segmentation in the two study regions, moving beyond national-level approaches to account for the internal complexity of both labor markets and their connection with historical conditions of capitalist reproduction in the global South.

## 2 Labor markets, segmentation and territories

From an institutionalist economic perspective, neoclassical equilibrium between labor supply and demand is simply impossible (Neffa, 2007). The labor market is viewed as a complex and conflictual social space (Solow, 1990; Pries, 2003), where information circulates in a fragmented manner. This limits access to opportunities, because labor markets are segmented into different groups with mobility barriers (Piore, 1975; Kerr, 1985; Neffa, 2008; Fernández Huerga, 2010; Bensusán Areous, 2022). The literature distinguishes a group of primary segments with high wages and stability, and a secondary group with low income, instability, and low mobility (Piore, 1975; Kerr, 1985; Casado Izquierdo, 2013). Segmentation is linked to differences in working conditions, precariousness, contracts, and income (Piore, 1975; Casado Izquierdo, 2013) and is reproduced through the educational system and public policies (Martín and Khöler, 2005, p. 315).

In turn, labor markets are constituted by information flows in areas with diffuse but demarcable geographic boundaries, where workers offer their services and companies purchase them. Commuting is defined as the movement between residence and workplace (Tolbert and Killian, 1987; Casado Díaz, 2000; Manzanares, 2012). Ultimately, territories integrate elements such as work, residence, services, and infrastructure, reducing the need for travel (Casado Díaz and Coombes, 2011).

The expansion of geographically localized labor markets depends on transportation costs, land use, production location, economic growth, and development policies—that is, business, institutional, and social factors. Labor markets are thus located in territories where people work, reside, and access services, building social and community networks (Berdegué et al., 2011), where gender and racial inequalities influence labor and migration mobility, shaping specific class structures and trajectories (Massey, 1984; Sánchez, 2003).

In Chile and Latin America, the neoliberal model and the end of the developmentalist matrix altered class structures, salary conditions, and the quality of employment, as well as the relationship between work, unemployment, and poverty (Portes and Hoffman, 2003; León and Martínez, 2001; Ruiz and Boccardo, 2015; Fuentes and Hernando, 2019). The profound metamorphoses of capitalism generated a varied and fragmented range of labor groupings (Antunes, 2001), making the boundaries between dependent and independent, fixed and flexible, direct or subcontracted activities increasingly porous (Gálvez, 2001; Palomino, 2001). Precariousness was consolidated globally (Standing, 2013), and in Chile it is evident as a “trans-class” phenomenon, even affecting qualified fractions (Blanco and Julián, 2019). Thus, the unemployed, underemployed, and underutilized proliferate within the new labor regimes.

At the national level, the middle and working classes are concentrated in the private sector, especially in commerce and services (León and Martínez, 2001; Portes and Hoffman, 2003; Ruiz and Boccardo, 2015). Despite higher levels of education, social mobility remains limited (Wormald and Torche, 2004), while tertiary activities are highly precarious and informal (Weller, 2004; Blanco and Julián, 2019). This questions the thesis of Chilean social mesocratization, revealing a persistent presence of manual classes (Pérez, 2018). However, this must be analyzed from the perspective of geographical inequalities in labor, since the middle classes are concentrated in large cities and are reduced in isolated and rural territories (Lufin and Atienza, 2009; Mac-Clure et al., 2014). In recent decades, there has been a growing outsourcing of intermediate cities, even losing their specialization in certain primary activities (Fuentes et al., 2017).

In Chile, large cities such as Santiago and Valparaíso have diverse economic sectors, while isolated territories specialize in a few sectors, and intermediate cities are incipiently expanding their services, trade, and intermediation activities, and, to a lesser extent, manufacturing industries (Escolano et al., 2016; Fuentes et al., 2017). This contributes to an unequal employment distribution between regions, with dual markets and internal variability in major cities. In rural territories—particularly in areas dominated by primary or extractive economies—precariousness, flexibility, and informality are consolidated (Valdés et al., 2014; Julián-Vejar and Alister, 2018; Blanco et al., 2020). These differences generate disparities in productive matrices, labor markets, and access to globalization (ECLAC, 2009; Escolano et al., 2016).

## 3 Intra and interregional comparison

The processes described above are not homogeneous across the country. The evolution of capital accumulation promotes the articulation of differentiated territorial dynamics, forcing local economies to restructure their labor systems in a context marked by intensified inter- and intra-territorial competition (Brandão, 2010). More broadly, interregional competition and specialization reveal internal and external dynamics that shape distinct forms of labor market structuring. In this sense, comparing the labor typologies of the Antofagasta and Araucanía regions makes it possible to identify distinct patterns and trends in the structure of employment, as well as relevant similarities in the configuration of their labor markets.

To achieve this, a dual analytical strategy is adopted for a comprehensive understanding of labor dynamics. On the one hand, intraregional analysis serves to characterize the labor structure within each region, taking into account its provinces and internal territorial dynamics. On the other hand, interregional analysis aims to compare the labor structure between both regions, emphasizing the differences and similarities in the composition and distribution of labor groups (Table 1).

**Table 1:** Scope of the inter and intra-regional analyses of this study.

Interregional analysis	Intra-regional analysis
Construction of comparable labor typologies for each region. Comparison of the structural factors of regional labor markets. Identification of similarities and differences in regional job profiles.	Distribution of job types in each province of both regions. Characterization of socioeconomic, demographic and labor variables of the labor segments of each region. Identification of the sectoral concentration of specific labor groups.

Source: Prepared by the authors.

Intraregional analysis enables a deeper understanding of the internal structure of each region, revealing significant territorial differences in the distribution of labor segments. These can be distinguished according to criteria such as formality, income levels, or the most significant economic sectors. For example, it is possible to investigate whether a labor group is distributed homogeneously (concentrated in a few sectors) or diversely (present in multiple economic sectors) within a single region. It is also possible to examine the existence of significant wage differences between workers in the same group depending on their province of residence, among other factors.

Interregional analysis seeks to establish contrasts between Antofagasta and La Araucanía regarding the structure of their labor markets. This comparison reveals not only divergences in the configuration of labor groups, but also potential structural similarities in terms of segmentation, informality, and sectoral specialization.

## 4 Methodology: database, variables and analysis techniques

The data source used was the 2022 version of the National Socioeconomic Characterization Survey (CASEN). The following variables were used for the indicators:

The method used in this study for the generation of segments and profiles has been developed and applied in various contexts (Blanco, 2019; Blanco and Julián, 2019; Blanco et al., 2020; Blanco et al., 2022, among others) as part of a research program on the geographical variability of local labor configurations (Blanco, 2019).

In summary, the entire procedure for constructing job profiles can be summarized as follows:

1. The first step was to apply multiple correspondence analysis to all variables in Table 2. This allows for the analysis of associations between categorical variables and the graphic representation of their relationships in a reduced dimensional space, facilitating the identification of patterns and underlying groupings in the data structure. The contributions of the variables to the factorial dimensions were examined, allowing for a theoretical interpretation of labor market factors in both regions. Additionally, perceptual maps were developed to visualize the distribution of occupational groups in the factorial space. The resulting coordinates were also entered into the database.
2. In a second step, the factor scores are used in a k -means cluster analysis, establishing the occupational clusters. Although the variables analyzed are qualitative, the method does not incorporate hierarchical cluster analysis. Instead, two new variables were stored in the database, linked to the case scores on the two factorial dimensions of the MCA. These two quantitative variables (the factorial scores on axes 1 and 2) are used in the creation of the labor typology through k-means clustering. It is a combination of MCA, factorization, factorial scores, and the use of the latter in the segmentation and delineation of labor profiles. This procedure makes it possible to construct a “typology” through cluster analysis, as well as to identify the factorial dimensions that synthesize the information into two axes of a Cartesian plane. The latter represents a graphic approximation of the structural axes of the labor market in both regions.
3. Additionally, the final number of class profiles is defined using one-way ANOVA, analyzing different solutions for reducing within-group variance and increasing between-group heterogeneity. Along with the analysis of intra-categorical variance, the final number of groups was determined based on criteria of clarity and interpretive parsimony.
4. The final step is the characterization of segments and classes through a broad range of labor, socioeconomic, demographic, and territorial variables, a procedure through which it will be possible to name and identify characteristics of each aggregate produced.

**Table 2:** Variables and categories for the construction of the labor typology.

Variable	Categories
Sex	1) Man; 2) Woman.
Ethnicity	1) Belongs; 2) Does not belong.
Nationality	1) Chile; 2) Chile and another country; 3) another country.
Province	Antofagasta: 1) Antofagasta; 2) El Loa; 3) Tocopilla. The Araucanía: 1) Cautín; 2) Malleco.
Deciles of earned income (in Chilean pesos)	1) < = 200,000; 2) 200,001 – 322,000; 3) 322,001 – 400,000; 4) 400,001 – 450,000; 5) 450,001 – 506,667; 6) 506,668 – 608,333; 7) 608,334 – 800,000; 8) 800,001 – 1,000,000; 9) 1,000,001 – 1,600,000; 10) 1,600,001+.
International Standard Classification of Occupations (ISCO)	1) Members of the executive and legislative branches and management staff of public administration and businesses; 2) Professionals, scientists, and intellectuals; 3) Mid-level technicians and professionals; 4) Office workers; 5) Service workers and vendors in shops and markets; 6) Farmers and skilled agricultural and fishing workers; 7) Officials, operators, and artisans in mechanical arts and other trades; 8) Plant and machine operators and assemblers; 9) Unskilled workers;
International Classification of Status in Employment (ICSE)	1) Employer; 2) Self-employed; 3) Public sector employee or worker (Central or Municipal Government); 4) Public enterprise employee or worker; 5) Private sector employee or worker; 6) Live-in domestic service; 7) Outdoor domestic service; 8) Unpaid family member.
International Standard Industrial Classification of All Economic Activities (ISIC).	1) Agriculture, Livestock, Forestry and Fishing; 2) Mining and Quarrying; 3) Manufacturing; 4) Electricity, Gas, Steam and Air Conditioning Supply; 5) Water Supply; 6) Wastewater Disposal, Waste Management and Remediation; 7) Construction; 8) Wholesale and Retail Trade; 9) Motor Vehicle and Motorcycle Repair; 10) Transportation and Warehousing; 11) Accommodation and Food Service Activities; 12) Information and Communications; 13) Financial and Insurance Activities; 14) Real Estate Activities; 15) Professional, Scientific and Technical Activities; 16) Administrative and Support Service Activities; 17) Public Administration and Defense; 18) Compulsory Social Security Schemes; 19) Teaching; 20) Human Health Care and Social Assistance Activities; 21) Artistic, Entertainment, and Recreational Activities; 22) Other Service Activities; 23) Activities of Households as Employers; 24) Undifferentiated Activities of Households as Producers of Goods and Services; 25) Activities of Extraterritorial Organizations and Bodies.
Written employment contract.	1) Yes; 2) No.
Type of contract	1) Indefinite term; 2) Fixed term; 3) Per work or task.
Affiliation to the pension system	1) Yes; 2) No.
Health	1) Public System (FONASA); 2) ISAPRE (private health insurance institution); 3) Armed Forces and Law Enforcement; 4) None (private); 5) Other system.
Did you contribute to a pension system last month?	1) Yes; 2) No.

Source: CASEN 2022.

## 5 Interregional analysis

### 5.1 Typologies of labor segmentation

The factor models for both regions are structured by two dimensions with high Cronbach’s alphas, indicating high internal reliability (Appendix). In absolute terms, the total variance explained in the Antofagasta region model is slightly higher than in the Araucanía region model (6.759 versus 6.531). This is not a significant difference, so it is possible to maintain that both regions share a similar factor structure in terms of explained variance. In terms of inertia, both models explain between 56.3% (Antofagasta) and 56% (Araucanía) of the total variance. All this evidence suggests that we are dealing with stable factor structures and adequate overall internal consistency.

When constructing the final solution for each regional labor typology, differences began to be observed in relation to the interpretation and final physiognomy of the groups. In the case of Antofagasta, the final solution for the labor typology resulted in the identification of six groups (Table 3) that reflect the dynamics of the labor market in the region, highlighting the specificities of the occupational structure and labor segmentation in this territory. The groups in the Antofagasta labor typology are as follows:

- **Specialized Private sector workers:** A group where employment is predominantly in the private sector, with a high presence of professionals, technicians, and managers, and considerable links to mining and specialized sectors.
- **Operational Private sector workers:** Comprised of private sector workers with operational and commercial functions, with a high incidence of basic occupations, operators, and support staff.
- **High-ranking public Officials:** A group composed primarily of public officials, highlighting professional and technical profiles who hold positions of greater responsibility in state administration, the public education system, and health services.
- **Operational Public sector workers:** Comprised of public sector officials and workers in entry-level or lower-level positions, where there is a predominance of operators and service workers within the administration.
- **Various self-employed workers:** It groups self-employed activities, with a heterogeneous occupational structure that includes service workers, artisans, and operators, present in various economic sectors.
- **Domestic service workers:** A group comprised of services provided in the home, with a predominance of basic domestic and care work.

**Table 3:** Typology of labor profiles in Antofagasta.

Occupational profiles	Count	Percentage
Specialized private sector workers	141,940	44.1%
Operational private sector workers	92.630	28.8%
High-ranking public officials	12.592	3.9%
Operational public sector workers	6.148	1.9%
Various self-employed workers	61,925	19.2%
Domestic service workers	6.576	2.0%
Total	321.811	100.0%

Source: Prepared by the authors using CASEN 2022 data.

Antofagasta’s labor market is dominated by private sector employment, reflecting a landscape heavily influenced by the mining and related industries. The private sector segment is structured in a dual manner, with Specialized private sector workers (44.1%) in mining and related activities, along with an Operational profile Private sector workers (28.8%) who support this industry through occupations related to machine operators, administrative assistants, sales personnel, and other employees in commercial and service sectors.

On the other hand, the presence of self-employment suggests the existence of a complementary economy that facilitates the absorption of part of the labor demand not covered by the more formal sectors. However, the presence of self-employment or self-employment only reaches a fifth of the employed population (19.2%). These self-employed segments linked to informal or precarious micro-business activities are primarily located in commerce, transportation, and other services, and may be associated with high labor turnover and dependence on complementary economic activities.

It is also worth highlighting the low proportion of high- and low-level public officials (5.8%) in relation to the working population, reinforcing the thesis of the preponderance of the private sector in terms of employment within the region’s economic structure. Finally, the Domestic service workers shows low presence (2.0%), a matter that could be due to high relative incomes from other sectors that reduce the supply of workers in this area.

In the case of the Araucanía region, a solution of eight groups has been established, reflecting the diversity and segmentation of the labor market and the region’s unique socioeconomic and productive characteristics. Thus, the labor typology groups for Araucanía are as follows:

- **High-ranking public Officials:** Professionals and technicians who work primarily in the public sector and state-owned enterprises. Their presence is concentrated mainly in areas such as public education, public health services, and municipal administration.
- **Skilled operational Private sector workers:** Mid-level operators and technicians with a strong presence in the manufacturing and construction sectors. Despite their relatively higher qualifications, these workers are prone to precarious work, lack protection, and lack employment contracts.
- **Professionals and technicians in the public and private sectors:** A group of employees with technical and professional training in sectors such as education, health, and public administration, both public and private.
- **Operational Private sector workers in construction and manufacturing:** A group of employees in the construction and manufacturing sectors, with a high presence of operators and basic occupations.
- **Operational private sector workers in trade and basic services:** A group of low-skilled activities in the private sector, primarily in commerce.
- **Self-employed Traders and vendors:** Mostly self-employed workers in various activities, with a predominance of retail trade.
- **Self-employed agricultural and construction workers:** People who independently carry out agricultural activities and manual trades.
- **Domestic service workers:** Group with a high concentration of elementary occupations linked to domestic and care work.

According to the data in Table 4, the employment distribution in the Araucanía region presents a diverse and segmented labor structure representative of the territory’s socioeconomic and productive characteristics. Indeed, there is a labor market with a strong presence of public employment and a diversification in the private sector, where self-employment and the agricultural sector play a significant role in shaping the regional employment structure.

**Table 4:** Typology of labor profiles in La Araucanía.

Occupational profiles	Count	Percentage
High-ranking public officials	89,726	22.1%
Skilled operational private sector workers	5.881	1.5%
Professionals and technicians in the public and private sectors	90,576	22.4%
Operational private sector workers in construction and manufacturing	52,706	13.0%
Operational private sector workers in trade and basic services	6.413	1.6%
Self-employed traders and vendors	83,907	20.7%
Self-employed agricultural and construction workers	54,467	13.4%
Domestic service workers	21,549	5.3%
Total	405.225	100.0%

Source: Prepared by the authors using CASEN 2022 data.

The sum of Professionals and technicians in the public and private sectors (22.4%) and High-ranking public Officials (22.1%) make up almost half of the workforce, demonstrating a concentration of jobs around public administration and state services. This reflects the importance of the state apparatus in the region.

Regarding diversification in private employment, there is a division between a very small fraction of skilled operational private sector workers (1.5%) and Operational private sector workers in construction and manufacturing (13.0%), as well as Operational private sector workers in trade and basic services (1.6%). This suggests that, while there is a niche of operational workers in productive sectors, the representation of these groups is significantly lower compared to the public sector, a situation completely opposite to the case in Antofagasta.

Self-employment, for its part, also presents significant interregional differences. Two segments stand out: Self-employed traders and vendors (20.7%), and self-employed agricultural and construction workers (13.4%). This reflects the precariousness of self-employment and the persistent presence of traditional productive activities, which remain important pillars of the Araucanía economy.

Domestic group service workers (5.3%) indicates a demand that reaches double that observed in Antofagasta, an issue that could be related to family dynamics and forms of subjection linked to the extension of care work.

### 5.2 Structural factors of regional labor markets

**CHART 1:**
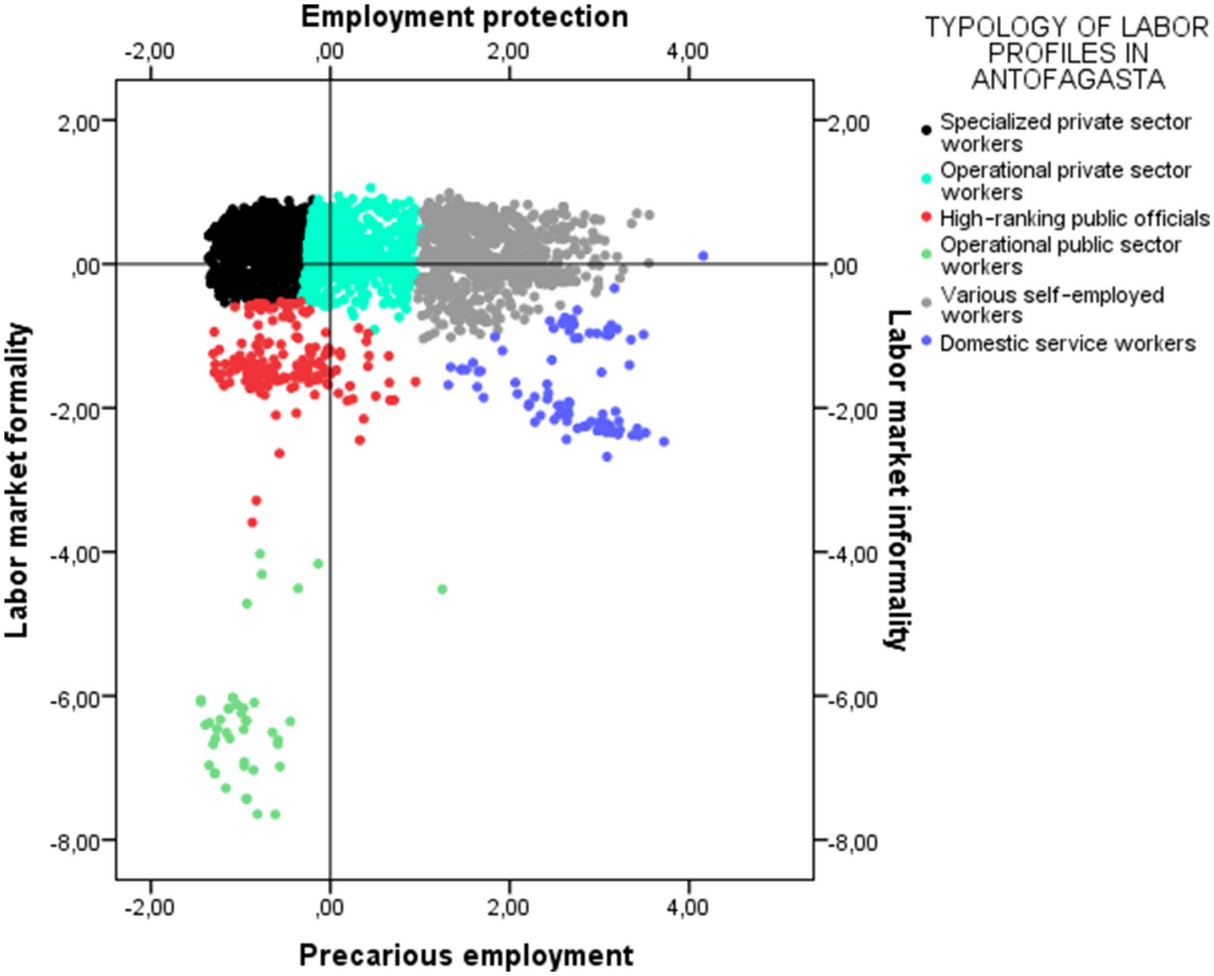
Job typology in the space of structural factors of the labor market in the Antofagasta region. Source: Prepared by the authors using CASEN 2022 data.

**CHART 2:**
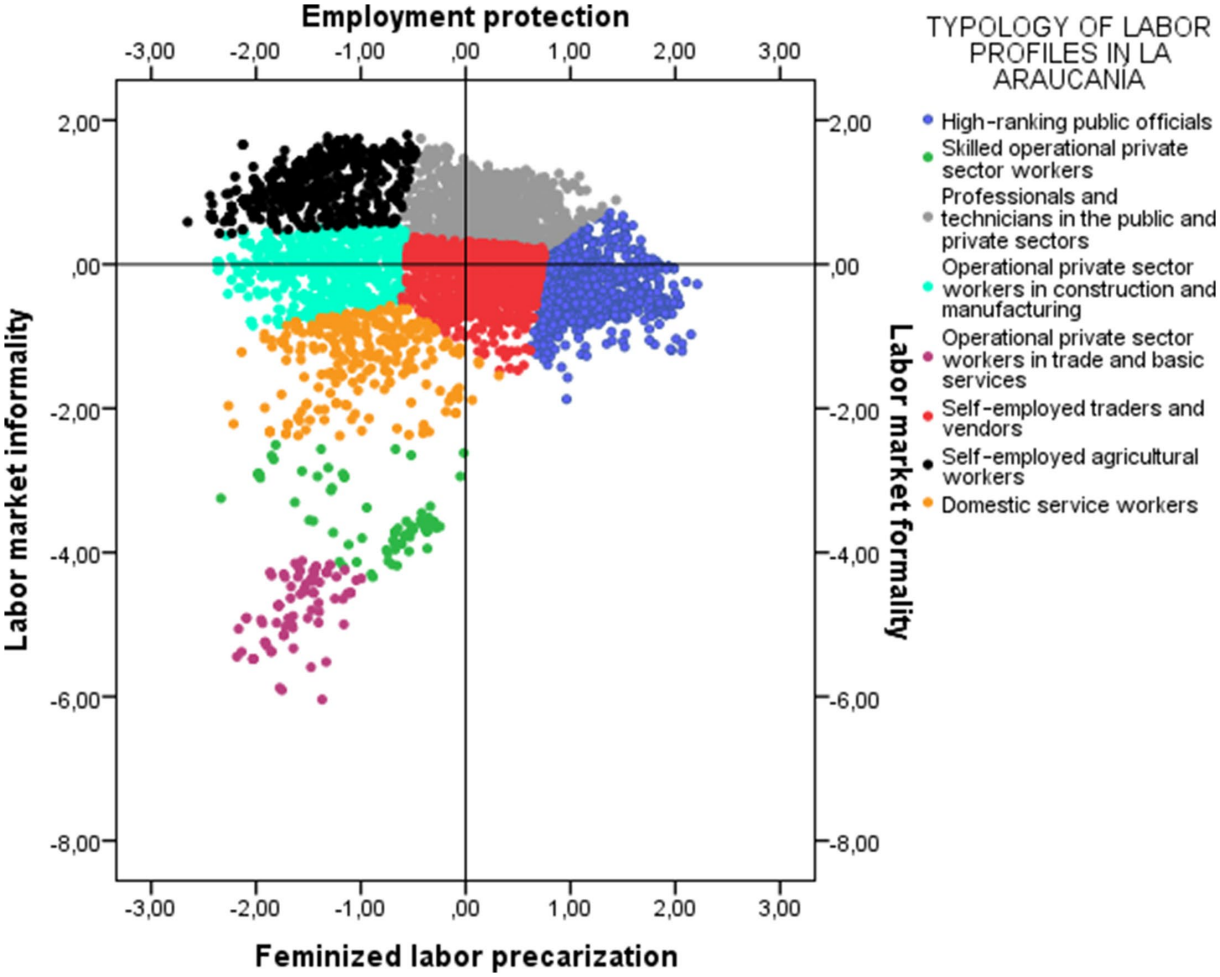
Job typology in the space of structural factors of the labor market in the Araucanía region. Source: Prepared by the authors using CASEN 2022 data.

Charts 1, 2 are visual representations of the distribution of occupational groups in their respective regional multidimensional spaces. This provides insight into the structure and distribution of occupational groups, facilitating the analysis of similarities, differences, proximities, and distances. It also provides an overview of the factors or dimensions that shape the distribution of employment conditions and situations. In other words, both figures can be interpreted as visual representations of regional employment spaces, structured by their main factors.

In Antofagasta, the structural factors space of the labor market is established as follows:

- **Axis 1: Labor market Formality/Informality:** This factor reflects a gradient that oscillates between informality and formality. On the positive side are situations linked to the absence of a formal contract, typical of self-employment. Conversely, jobs with formalized labor ties are located on the negative side of the axis. The main inertias^1^ show that Dimension 1 distinguishes between formal job placements—with a written contract, pension affiliation, and permanent jobs (negative values)—and forms of self-employment or jobs without social protection (positive values).
- **Axis 2: Employment protection/Precarious Employment:** This factor ranges from low-protection employment conditions to more stable and secure conditions, primarily linked to military and civil service positions, along with their respective pension systems. At the negative end are jobs with precarious working conditions and weak coverage in their social security systems. In contrast, the highest-weighted categories correspond to the Armed Forces, civil service, and mandatory pension systems, concentrated at the negative end of the scale.

In the case of the Araucanía region, the structural factors of the labor market are organized as follows:

- **Axis 1: Labor market Formality/Informality:** This factor represents a continuum ranging from jobs with high informality to positions associated with greater formalization. This axis groups together those categories linked to self-employment or jobs with poor contracts, compared to occupations in sectors with formal contracts and better pay. This factor is structurally very similar to the first dimension observed in Antofagasta.
- **Axis 2: Employment Protection/Feminized labor precarization:** This factor is related to various processes of feminization of employment, particularly in activities related to domestic work. Unlike what was observed in Antofagasta, in the Araucanía region, the main categories associated with the inertia of this dimension are women and the branch of Household Activities as employers and domestic service outside the home. In contrast, men are at the opposite extreme, with a lower association with this dimension.

Thus, the Antofagasta and Araucanía regions present a similarity in their first factorial axis, corresponding to the differentiation between formal and informal employment. This means that both regions distinguish between jobs with a written contract, pension affiliation, and stability versus self-employment and jobs lacking social protection.

The main divergence between the two regional configurations lies in the second dimension. Indeed, in Antofagasta, the second dimension reinforces the differences between unstable working conditions and low pension coverage compared to stable jobs in the public sector and the Armed Forces. In contrast, in La Araucanía, the second dimension reflects a process of feminized precarization, where the main inertia is linked to domestic service activities, evidencing a pattern of occupational segmentation with strong gender components.

It is important to clarify that the factorial axes are not predetermined, but rather are defined based on the combinations of categories that explain the greatest inertia in each data set. In other words, the multidimensional graphs do not share the same axes, but rather represent different theoretical dimensions, which is explained by the fact that they reflect labor markets with distinct structural characteristics. Both graphs represent factorial spaces that synthesize various theoretical dimensions and more effectively summarize the information available in each region. Far from constituting an inconsistency, this difference demonstrates that local labor markets have unequal structures and that factorial configurations must be interpreted considering these territorial specificities.

Thus, while in Antofagasta and La Araucanía the first factorial dimension is related to different situations of formality and informality, the second axis reveals significant structural differences between the two labor markets. That is, while in Antofagasta a continuum is defined ranging from protection to precariousness without discrimination by sex, in La Araucanía this dimension allows us to observe a specifically female precariousness. In other words, the precariousness of employment in La Araucanía is especially relevant for working women living in the region.

### 5.3 Job typologies, territoriality and general characteristics

As a result, although both labor markets have formal and informal segments, their structures differ. Antofagasta is characterized by a predominance of private employment linked to the mining and commercial sectors, while in La Araucanía, the public sector and occupations linked to traditional and agricultural activities predominate.

In fact, the distribution of job types in Antofagasta and La Araucanía presents marked contrasts that reflect the structural differences of their economies. In Antofagasta, the mining and quarrying sector is confirmed to be the predominant one, accounting for 32.7% of the specialized private sector workers and 15.9% of the total workforce, demonstrating the centrality of mining (Table 5). In contrast, in La Araucanía, mining is insignificant, while agriculture, livestock, forestry, and fishing account for 12.7%, highlighting the region’s more agricultural character (Table 6). Similarly, in Antofagasta, commerce represents 17.7% of the total workforce, with a strong presence of various self-employed workers (34.5%) (Table 5). In La Araucanía, vehicle trade and repair is the most representative sector (17.1%), with a high percentage of self-employed workers. Traders and vendors (31.5%) (Table 6).

**Table 5:** Percentage distribution of economic branches in the labor segments of the Antofagasta region.

ISIC economic sectors	Specialized private sector workers	Operational private sector workers	High-ranking public officials	Operational public sector workers	Various self-employed workers	Domestic service workers	Total
Agriculture, livestock, forestry and fishing	0.2%	0.5%	0.0%	0.0%	7.4%	0.1%	1.6%
Mining and quarrying	32.7%	5.2%	0.0%	0.0%	0.1%	0.0%	15.9%
Manufacturing industries	8.0%	7.8%	0.6%	0.0%	10.1%	0.0%	7.8%
Supply of electricity, gas, steam and air conditioning	2.0%	0.1%	0.0%	0.0%	0.0%	0.0%	0.9%
Water supply; wastewater disposal, waste management, and decontamination	1.7%	0.5%	0.0%	0.0%	0.6%	0.0%	1.0%
Construction	4.8%	12.3%	0.0%	0.0%	12.2%	0.0%	8.0%
Wholesale and retail trade; repair of motor vehicles and motorcycles	8.3%	25.5%	0.0%	0.0%	34.5%	2.6%	17.7%
Transport and storage	6.1%	9.1%	1.2%	0.0%	9.5%	0.0%	7.2%
Accommodation and catering activities	1.1%	10.9%	0.0%	0.0%	8.2%	0.0%	5.2%
Information and communications	1.2%	0.8%	0.0%	0.0%	0.5%	0.0%	0.9%
Financial and insurance activities	2.0%	0.1%	0.0%	0.0%	0.0%	0.0%	0.9%
Real estate activities	0.3%	1.2%	0.0%	0.0%	0.6%	0.0%	0.6%
Professional, scientific and technical activities	4.7%	4.0%	0.0%	0.0%	0.7%	0.0%	3.3%
Administrative and support service activities	5.0%	7.5%	0.0%	0.0%	1.9%	0.0%	4.7%
Public administration and defense; mandatory social security plans	0.0%	0.0%	71.7%	98.2%	0.1%	0.2%	4.7%
Teaching	13.3%	5.4%	14.1%	1.8%	0.3%	0.0%	8.1%
Human health care and social assistance activities	8.0%	3.2%	11.0%	0.0%	0.8%	0.0%	5.0%
Artistic, entertainment and recreational activities	0.3%	1.2%	0.7%	0.0%	0.9%	0.0%	0.7%
Other service activities	0.4%	4.1%	0.0%	0.0%	10.7%	3.0%	3.5%
Activities of households as employers; undifferentiated activities of households as producers of goods and services	0.0%	0.5%	0.6%	0.0%	0.8%	94.2%	23%
Activities of extraterritorial organizations and bodies	0.0%	0.0%	0.0%	0.0%	0.0%	0.0%	0.0%
Total	100.0%	100.0%	100.0%	100.0%	100.0%	100.0%	100.0%

Source: Prepared by the authors using CASEN 2022 data.

**Table 6:** Percentage distribution of economic branches in the labor segments of the Araucanía region.

ISIC economic sectors	High-ranking public officials	Skilled operational private sector workers	Professionals and technicians in the public and private sectors	Operational private sector workers in construction and manufacturing	Operational private sector workers in trade and basic services	Self-employed traders and vendors	Self-employed agricultural and construction workers	Domestic service workers	Total
Agriculture, livestock, forestry and fishing	0.7%	0.0%	13.3%	23.7%	0.0%	13.4%	24.7%	7.1%	12.7%
Mining and quarrying	0.4%	0.0%	2.0%	0.0%	0.0%	0.2%	0.0%	0.0%	0.6%
Manufacturing industries	0.8%	0.0%	17.4%	6.2%	0.0%	3.5%	18.4%	0.6%	8.1%
Supply of electricity, gas, steam and air conditioning	0.1%	0.0%	1.4%	0.0%	0.0%	0.2%	0.0%	0.0%	0.4%
Water supply; wastewater disposal, waste management, and decontamination	0.0%	0.0%	2.2%	0.3%	0.0%	1.5%	0.0%	0.6%	0.9%
Construction	0.2%	0.0%	25.9%	5.6%	0.0%	5.2%	31.9%	1.2%	12.0%
Wholesale and retail trade; repair of motor vehicles and motorcycles	1.5%	0.0%	9.7%	39.4%	0.0%	31.5%	10.2%	29.9%	17.1%
Transport and storage	0.4%	0.0%	11.5%	23%	0.0%	1.3%	7.9%	0.2%	4.3%
Accommodation and catering activities	0.4%	1.9%	0.3%	6.3%	0.0%	12.1%	0.2%	27.0%	5.0%
Information and communications	1.5%	0.0%	2.8%	0.0%	0.0%	0.8%	0.2%	0.0%	1.2%
Financial and insurance activities	2.0%	0.0%	0.1%	0.0%	0.0%	0.5%	0.0%	0.0%	0.6%
Real estate activities	0.2%	0.0%	1.0%	0.3%	0.0%	0.4%	0.1%	0.0%	0.4%
Professional, scientific and technical activities	4.9%	1.1%	4.0%	0.9%	0.0%	3.0%	0.4%	0.3%	2.8%
Administrative and support service activities	0.4%	4.6%	1.6%	3.3%	0.0%	5.2%	3.4%	2.0%	2.6%
Public administration and defense; mandatory social security plans	22.4%	0.0%	1.0%	0.0%	0.0%	2.9%	0.0%	1.0%	5.9%
Teaching	35.5%	0.0%	1.4%	0.1%	0.0%	7.5%	0.0%	1.7%	9.8%
Human health care and social assistance activities	27.8%	1.8%	1.4%	1.3%	0.0%	6.3%	0.0%	4.6%	8.2%
Artistic, entertainment and recreational activities	0.5%	0.0%	1.7%	1.1%	0.0%	1.1%	0.0%	0.5%	0.9%
Other service activities	0.3%	0.0%	1.5%	8.8%	0.0%	3.4%	2.6%	5.3%	2.9%
Activities of households as employers; undifferentiated activities of households as producers of goods and services	0.1%	90.7%	0.0%	0.2%	100.0%	0.0%	0.0%	18.0%	3.9%
Activities of extraterritorial organizations and bodies	0.0%	0.0%	0.0%	0.0%	0.0%	0.0%	0.0%	0.0%	0.0%
Total	100.0%	100.0%	100.0%	100.0%	100.0%	100.0%	100.0%	100.0%	100.0%

Source: Prepared by the authors using CASEN 2022 data.

Regarding public administration, in Antofagasta the High-ranking public officials and the Operational Public sector workers are concentrated in this sector (71.7 and 98.2%, respectively) (Table 5), while in La Araucanía the High-ranking public officials are concentrated in public administration, education and health (22.4, 35.5 and 27.8%) (Table 6). As mentioned above, another relevant difference is the higher proportion of Domestic service workers in La Araucanía compared to Antofagasta, reflecting the precariousness of employment in the southern region.

The productive and labor market structures of both regions are structurally different. Antofagasta has a more urban profile, with a high concentration of employment in the extractive, service, and related sectors. In contrast, La Araucanía shows a productive and labor market in transition, characterized by a more proportional distribution between traditional and urban agricultural activities, with commerce and construction as two relevant economic sectors in both urban and rural areas. In La Araucanía, this greater diversification of the urban–rural duality allows rural dynamics to coexist with urbanization processes, unlike the markedly urban-industrial character of Antofagasta, with its small and isolated rural populations.

Antofagasta shows a marked concentration of urban areas across all six job profiles, with almost 97% of cases located in urban areas (Table 7). This reflects the region’s high urbanization, driven by accumulation processes linked to mining and related sectors. Antofagasta is structured around mining, industry, commerce, and services, with a high presence of independent workers, while La Araucanía is based on agriculture and commerce, with a strong presence of professionals in public administration, education, and health.

**Table 7:** Percentage distribution of urban and rural areas in the labor segments of Antofagasta and La Araucanía.

Occupational profiles		Area		
		Urban	Rural	Total
Typology of labor profiles in Antofagasta	Specialized private sector workers	98.6%	1.4%	100%
	Operational private sector workers	96.4%	3.6%	100%
	High-ranking public officials	97.7%	23%	100%
	Operational public sector workers	100.0%	0.0%	100%
	Various self-employed workers	91.8%	8.2%	100%
	Domestic service workers	97.0%	3.0%	100%
	Total	96.6%	3.4%	100%
Typology of labor profiles in la Araucanía	High-ranking public officials	86.7%	13.3%	100%
	Skilled operational private sector workers	76.3%	23.7%	100%
	Professionals and technicians in the public and private sectors	79.4%	20.6%	100%
	Operational private sector workers in construction and manufacturing	65.7%	34.3%	100%
	Operational private sector workers in trade and basic services	82.5%	17.5%	100%
	Self-employed traders and vendors	80.2%	19.8%	100%
	Self-employed agricultural and construction workers	58.5%	41.5%	100%
	Domestic service workers	80.7%	19.3%	100%
	Total	76.7%	23.3%	100%

Source: Prepared by the authors using CASEN 2022 data.

Unlike the urban economic landscape of Antofagasta, La Araucanía demonstrates greater diversity in geographic distribution. Only 76.7% of workers live in urban areas, while 23.3% live in rural areas. This discrepancy is especially notable in segments such as Operational private sector workers in construction and manufacturing (65.7% urban compared to 34.3% rural) and self-employed agricultural workers, where rural workers reach 41.5%. On the other hand, while it is true that in La Araucanía the Professionals and technicians in the public and private sectors, as well as the High-ranking public officials, have a higher urban concentration, but they also record considerable percentages of cases residing in rural areas (20.6 and 13.3%, respectively). All of this allows us to contrast the agricultural and rural vocation of La Araucanía with the strong predominance of the urban economy in Antofagasta.

### 5.4 Interregional comparative synthesis

The comparative analysis of labor segmentation typologies in Antofagasta and La Araucanía reveals both structural similarities and substantive differences in the socio-occupational configuration of their labor markets. The factor models reveal structural similarities between both regional labor markets in terms of the distinction between formal and informal labor markets, protection, and precariousness. The factor solution shows that Antofagasta also links protection to the Public Administration and Defense sectors, while in La Araucanía, the feminization of labor is a structurally relevant segment of precariousness.

When analyzing the configuration of labor typologies, divergences emerge that respond to the productive and social specificities of each territory. Antofagasta presents a labor market dominated by the private sector and strongly influenced by mining and commerce, with a clear division between specialized and operational workers, in addition to a low participation of public employment and domestic work. In contrast, La Araucanía exhibits a more diversified and fragmented structure, where public employment has a considerable weight, self-employment is divided between commerce, construction, and agriculture, and domestic work is much more prevalent than in Antofagasta.

These differences are the result of Antofagasta’s position as a more specialized and technologically advanced mining enclave. La Araucanía, for its part, maintains a mixed economy with a strong presence of the state and traditional activities. Thus, it is possible to speak of a greater presence of state employment, while maintaining a more traditional productive structure that is less integrated into dynamic accumulation sectors.

## 6 Intraregional analysis

### 6.1 Local labor peculiarities

Labor segmentation theory establishes a fundamental duality between protected and unprotected groups in the labor market. Our findings show that this distinction can also be linked to territorial differences.

The urban–rural dualism refers to the existence of two distinct labor configurations, with little integration between them. This phenomenon is clearly observed in the Antofagasta region, where a distinctly urban production and labor model predominates, centered on salaried employment in the private sector, especially in specialized and operational profiles linked to mining, industry, commerce, and services. In contrast, in the region’s rural areas, self-employment prevails, reflecting a spatially and functionally fragmented labor structure.

**MAP 2:**
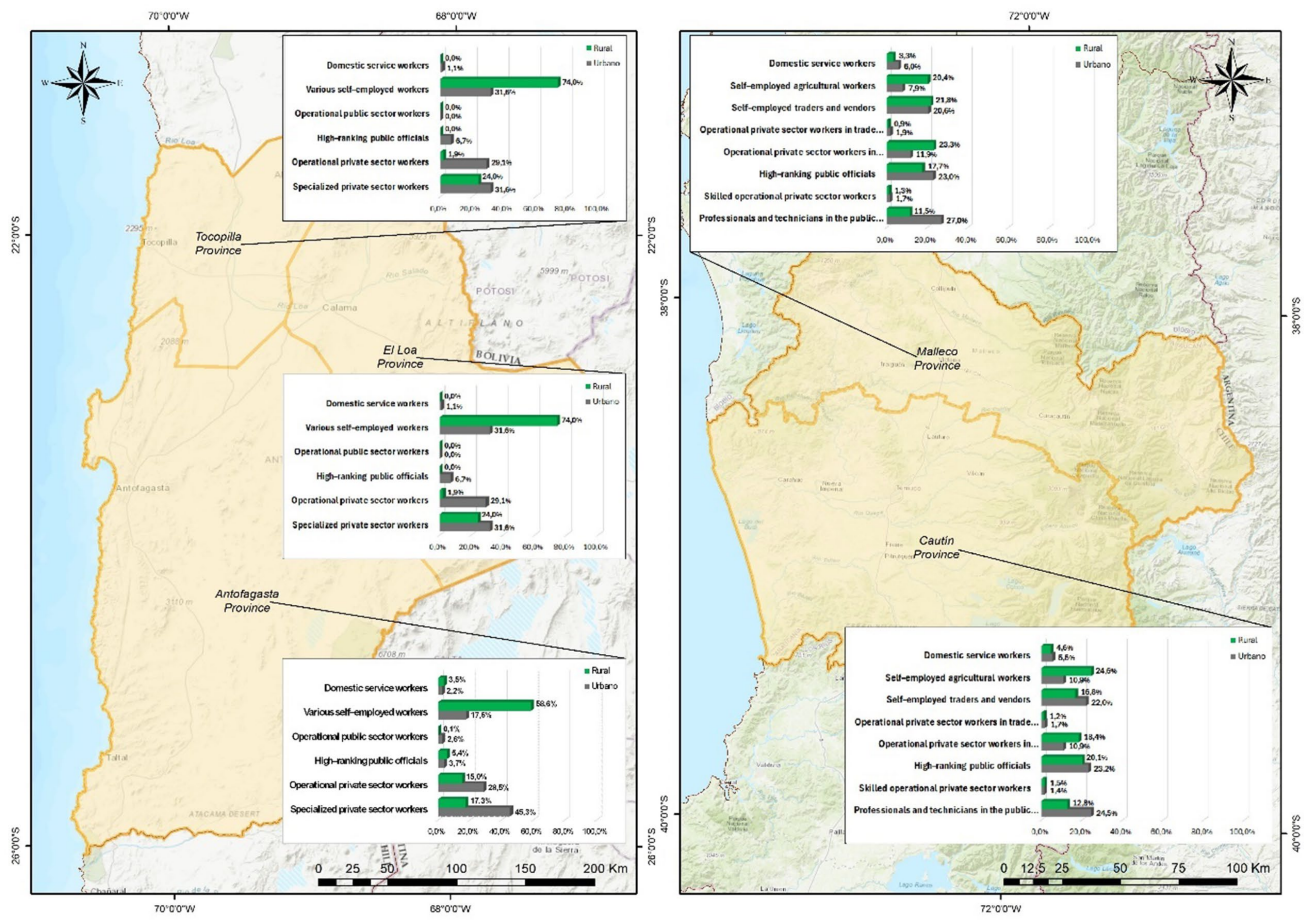
Distribution of job profiles in urban and rural areas, Antofagasta and La Araucanía regions. Source: Prepared by the authors using CASEN 2022 data. Antofagasta (*N* = 321,811), La Araucanía (*N* = 405,225).

In Antofagasta, urban employment is more formal, specialized, and predominantly private-sector, while rural employment is highly informal, reflected in the prevalence of self-employment. Regarding the latter, 74% of total rural employment in the province of Tocopilla shows high informality, corresponding to the various segment. Self-employed workers (Map 2). This means that rural employment in the Antofagasta region is residual and concentrated in self-employment with very low institutional coverage.

In contrast, the Araucanía region presents a more balanced distribution between urban and rural areas, reflected in a relatively homogeneous composition of labor segments within each area. This configuration suggests less territorial segmentation of employment (Map 2).^2^ In the region’s urban areas, professionals, senior public officials, operators, and self-employed workers in commerce predominate, while in rural areas, independent work predominates, along with a significant presence of public employment.

Regarding this last point, it is again evident that, unlike Antofagasta—where the public sector plays a marginal role in the employment structure—in La Araucanía, the public sector plays an important labor role, even in rural areas. This is evidenced by the balanced presence of high-ranking public officials in both urban and rural areas, which is sufficient proof to reaffirm the relevance of the public apparatus throughout the region, and not only in one type of territory.

In short, La Araucanía boasts a significant share of rural employment in various formal and informal sectors, including the State as an employer. Unlike the Antofagasta region, this configuration helps mitigate the urban–rural duality of the territory’s labor segmentation.

The above is confirmed by observing that, in the Antofagasta region, the Specialized Private sector workers predominate in both urban areas and the region as a whole (Table 8). In contrast, their presence decreases dramatically in rural areas, reflecting a specialization and functional concentration of skilled employment in urban areas.

**Table 8:** Percentage distribution of labor segments in urban and rural areas, Antofagasta and La Araucanía regions.

Occupational profiles		Urban	Rural	Regional total
Typology of work profiles in Antofagasta	Specialized private sector workers	45.0%	18.7%	44.1%
	Operational private sector workers	28.7%	30.6%	28.8%
	High-ranking public officials	4.0%	2.7%	3.9%
	Operational public sector workers	2.0%	0.0%	1.9%
	Various self-employed workers	18.3%	46.2%	19.2%
	Domestic service workers	2.1%	1.8%	2.0%
	Total	100%	100%	100%
Typology of work profiles in Araucanía	High-ranking public officials	25.0%	12.6%	22.1%
	Skilled operational private sector workers	1.4%	1.5%	1.5%
	Professionals and technicians in the public and private sectors	23.1%	19.8%	22.4%
	Operational private sector workers in construction and manufacturing	11.1%	19.1%	13.0%
	Operational private sector workers in trade and basic services	1.7%	1.2%	1.6%
	Self-employed traders and vendors	21.7%	17.5%	20.7%
	Self-employed agricultural and construction workers	10.2%	23.9%	13.4%
	Domestic service workers	5.6%	4.4%	5.3%
	Total	100%	100%	100%

Source: Prepared by the authors using CASEN 2022 data.

In the rural areas of the Antofagasta region, there is a high proportion of various self-employed workers, indicating a significant trend toward informality and self-employment in rural employment settings. Furthermore, the pattern linked to the low weight of public employment is repeated, where the share of high-ranking public officials and Operational Public sector workers are marginal in both areas, reflecting a low institutional or public service presence in those areas.

In the case of the Araucanía region, professional employment is concentrated in urban areas. Both professionals and technicians in the public and private sectors such as the Self-employed Traders and vendors represent similar percentages within the total of urban and rural territories (Table 8), showing that the formality and informality represented by both groups resides indistinctly in both types of territories.

In Araucanía, there is a much richer diversification of rural self-employment than in Antofagasta, highlighting the presence of self-employed workers. Agricultural and construction workers in rural areas, very different compared to urban areas. This reflects forms of subsistence or family production rooted in the rural context. Furthermore, there is a greater weight of rural operational work in sectors such as construction and manufacturing, with a higher proportion of Operational private sector workers in construction and manufacturing in rural areas than in urban areas, suggesting a greater presence of economic activities associated with local or regional production chains.

### 6.2 Demographic aspects of labor segmentation

Comparing the distribution of job types in both regions by sex (Table 9) reveals both similarities and differences. For example, in both regions, male participation is the majority in total employment (60% men and 40% women). However, gender segregation varies according to economic activity.

**Table 9:** Percentage distribution of men and women in labor segments, Antofagasta and La Araucanía regions.

Occupational profiles		Man	Women	Total
Typology of labor profiles in Antofagasta	Specialized private sector workers	71%	29%	100%
	Operational private sector workers	54%	46%	100%
	High-ranking public officials	36%	64%	100%
	Operational public sector workers	72%	28%	100%
	Various self-employed workers	54%	46%	100%
	Domestic service workers	2%	98%	100%
	Total	60%	40%	100%
Typology of labor profiles in la Araucanía	High-ranking public officials	40%	60%	100%
	Skilled operational private sector workers	1%	99%	100%
	Professionals and technicians in the public and private sectors	96%	4%	100%
	Operational private sector workers in construction and manufacturing	57%	43%	100%
	Operational private sector workers in trade and basic services	0%	100%	100%
	Self-employed traders and vendors	45%	55%	100%
	Self-employed agricultural and construction workers	92%	8%	100%
	Domestic service workers	15%	85%	100%
	Total	60%	40%	100%

Source: Prepared by the authors using CASEN 2022 data.

In Antofagasta, the Specialized private sector workers, as well as the Operational public sector workers, are dominated by men (71 and 72%, respectively), while women have a greater presence in the profile of the High-ranking public officials (64%) and in Domestic service workers (98%).

In La Araucanía, the gap is more extreme in some segments. Women are the majority in the skilled operational private sector workers (99%) and in the Operational private sector workers in trade and basic services (100% women). Men also show masculinized job segments, such as Professionals and technicians in the public and private sectors (96%) and self-employed agricultural workers (92%) are significantly masculinized. Similar to the case of Antofagasta, the High-ranking public sector among officials, the majority of cases are women (60%). In this sense, the segmentation observed in senior public sector positions is not due to gender factors, but rather to distinct patterns of territorial distribution.

In short, the difference between male-dominated and female-dominated segments can be associated with the productive structure. Antofagasta has a labor market more oriented toward industrial and specialized sectors, while La Araucanía reflects a strong gender divide in self-employment and service activities, with a lower female presence in high-level jobs. Indeed, gender gaps are accentuated in La Araucanía, with segments that are heavily feminized (domestic service and basic commerce), while others are almost exclusively male (in the agricultural and construction sectors).^3^

When comparing the employment typologies of both regions based on indigenous ethnicity (Table 10) and nationality (Table 11), structural differences in employment composition are observed. In Antofagasta, the indigenous population represents a smaller proportion in most job categories (13.4% in total), while in La Araucanía, the presence of cases belonging to ethnic groups is significantly higher (28.7%). However, despite these differences, both regions have a significant presence of cases belonging to ethnic groups.

**Table 10:** Percentage distribution of ethnic group membership in labor segments, Antofagasta and La Araucanía regions.

Occupational profiles		Belongs to an ethnic group		
		Forks	No	Total
Typology of labor profiles in Antofagasta	Specialized private sector workers	12.0%	88.0%	100%
	Operational private sector workers	13.5%	86.5%	100%
	High-ranking public officials	12.2%	87.8%	100%
	Operational public sector workers	15.7%	84.3%	100%
	Various self-employed workers	16.3%	83.7%	100%
	Domestic service workers	15.0%	85.0%	100%
	Total	13.4%	86.6%	100%
Typology of labor profiles in la Araucanía	High-ranking public officials	17.7%	82.3%	100%
	Skilled operational private sector workers	32.5%	67.5%	100%
	Professionals and technicians in the public and private sectors	26.3%	73.7%	100%
	Operational private sector workers in construction and manufacturing	38.0%	62.0%	100%
	Operational private sector workers in trade and basic services	25.5%	74.5%	100%
	Self-employed traders and vendors	30.5%	69.5%	100%
	Self-employed agricultural and construction workers	39.6%	60.4%	100%
	Domestic service workers	27.2%	72.8%	100%
	Total	28.7%	71.3%	100%

Source: Prepared by the authors using CASEN 2022 data.

**Table 11:** Percentage distribution of country of nationality in labor segments, Antofagasta and La Araucanía regions.

Occupational profiles		What is your country of nationality?			
		Chili	Chile and another country	Another country	Total
Typology of labor profiles in Antofagasta	Specialized private sector workers	90.8%	1.5%	7.7%	100%
	Operational private sector workers	76.3%	1.2%	22.6%	100%
	High-ranking public officials	98.2%	0.0%	1.8%	100%
	Operational public sector workers	94.9%	5.1%	0.0%	100%
	Various self-employed workers	69.9%	1.2%	28.9%	100%
	Domestic service workers	66.6%	0.7%	32.8%	100%
	Total	82.5%	1.3%	16.2%	100%
Typology of labor profiles in la Araucanía	High-ranking public officials	96.3%	1.9%	1.8%	100%
	Skilled operational private sector workers	97.3%	1.5%	1.2%	100%
	Professionals and technicians in the public and private sectors	96.8%	1.3%	1.9%	100%
	Operational private sector workers in construction and manufacturing	96.6%	1.1%	23%	100%
	Operational private sector workers in trade and basic services	95.2%	0.0%	4.8%	100%
	Self-employed traders and vendors	95.7%	1.2%	3.1%	100%
	Self-employed agricultural and construction workers	95.8%	1.7%	2.5%	100%
	Domestic service workers	93.2%	1.0%	5.9%	100%
	Total	96.1%	1.4%	2.5%	100%

Source: Prepared by the authors using CASEN 2022 data.

In terms of nationality, Antofagasta has a high proportion of migrant workers, particularly in low-skilled and informal sectors. For example, 32.8% of Domestic Workers service workers and 28.9% of various self-employed Workers are foreigners. In contrast, La Araucanía shows a predominantly Chilean composition^4^ (96.1%), with a low presence of foreign workers in all profiles, reflecting an employment structure less exposed to international migration.

In the case of the Domestic service workers, a particularity is observed in relation to foreign women in the Antofagasta region, where there is a significant presence of migrant women (32.8%). In this region, the Operational private sector workers and the various self-employed Workers are two other labor segments with a significant presence of foreigners.

Differences are also observed in the job hierarchy. In Antofagasta, the specialized private sector workers and the High-ranking public Officials have a low representation of indigenous and migrant workers, suggesting barriers to accessing more stable and remunerative jobs. In La Araucanía, the greater indigenous presence in operational and agricultural sectors reaffirms the Mapuche people’s historical relationship with rural productive activities (Table 10).

### 6.3 Income, qualifications and working conditions

**CHART 3:**
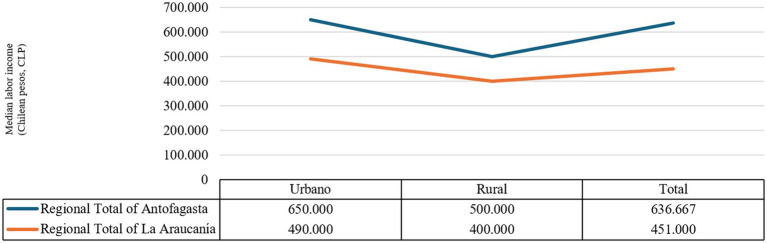
Median labor income (CLP) in the regional totals of Antofagasta and La Araucanía. Source: Prepared by the authors using CASEN 2022 data.

Median labor incomes In Antofagasta, the gaps in labor income inequality are higher than in La Araucanía, both in urban and rural areas (Chart 3).^5^ These urban–rural gaps in labor income inequality in both regions reveal contrasting patterns in the income distribution of some occupational profiles.

In both regions, the Domestic segment service the workers’ sector remains the lowest-income group, highlighting its structural precariousness. This is true regardless of the area (urban or rural). Meanwhile, despite its lower incidence in regional employment, the high-ranking public sector Antofagasta officials earn slightly higher incomes than their counterparts in La Araucanía. On the other hand, self-employed workers Traders and vendors in La Araucanía are the self-employed segment with the highest incomes, which indicates a labor market with fewer formal jobs, encouraging self-employment.

In general, urban areas concentrate higher incomes in a majority of occupational groups, especially those requiring higher qualifications or institutional integration. In fact, in Antofagasta, the only categories with higher incomes among rural residents are the Operational segments. Public sector workers and the various self-employed workers. In the case of La Araucanía, the skilled operational private sector workers and Operational private sector workers in trade and basic service are the labor segments that show higher rural incomes.

Thus, in La Araucanía, the profiles with the highest incomes among rural residents correspond to a type of activity linked to the operational private sector, both skilled and in basic services. In the case of Antofagasta, it is operational public officials and self-employment-related activities that show higher incomes in rural areas than in urban areas. These dynamics suggest that territorial location and occupational profile interact differently in each region, reproducing structural gaps in access to income.

On the other hand, in both regions, educational background is strongly correlated with the type of occupation, although there are notable differences between Antofagasta and La Araucanía (Table 12). In Antofagasta, the specialized private sector workers and the High-ranking public officials have a higher proportion of completed professional studies (30.2 and 43.0%, respectively), which reinforces the fact that this is a labor market oriented towards skilled and technical sectors. In contrast, the Operational private sector workers and those who work as Domestic Workers service Workers show a predominantly medium educational level (complete or incomplete).

**Table 12:** Distribution of labor segments by educational level, Antofagasta and La Araucanía.

Occupational profiles		Level of education														
		He does not know	Without formal education	Incomplete basic education	Complete basic	Incomplete humanistic media	Incomplete technical vocational training	Complete humanistic media	Complete professional technical level	Incomplete higher level technician	Complete higher level technician	Incomplete professional	Incomplete postgraduate degree	Complete professional	Complete postgraduate studies	Total
Typology of labor profiles in Antofagasta	Specialized private sector workers	1.4%	0.1%	0.6%	1.4%	2.9%	1.7%	18.4%	13.6%	3.9%	13.9%	7.5%	1.0%	30.2%	3.5%	100%
	Operational private sector workers	0.8%	0.7%	4.1%	4.3%	10.0%	2.7%	34.2%	20.6%	3.2%	6.6%	5.8%	0.1%	6.8%	0.1%	100%
	High-ranking public officials	0.0%	0.0%	1.8%	2.9%	4.3%	2.2%	12.7%	14.1%	2.1%	11.3%	3.5%	0.0%	43.0%	1.9%	100%
	Operational public sector workers	0.0%	0.0%	0.0%	0.0%	0.0%	0.0%	21.4%	5.0%	4.7%	38.5%	4.4%	3.6%	22.4%	0.0%	100%
	Various self-employed workers	0.6%	1.4%	10.3%	10.7%	14.2%	3.5%	30.3%	11.8%	3.7%	5.2%	4.6%	0.0%	3.8%	0.0%	100%
	Domestic service workers	2.5%	1.7%	6.9%	14.1%	10.4%	0.8%	45.8%	9.4%	1.7%	1.6%	1.5%	0.0%	3.5%	0.0%	100%
	Total	1.0%	0.5%	3.6%	4.3%	7.3%	23%	25.6%	15.0%	3.6%	10.2%	6.1%	0.5%	18.2%	1.6%	100%
Typology of labor profiles in la Araucanía	High-ranking public officials	0.7%	0.0%	0.8%	0.5%	0.7%	1.3%	10.8%	7.2%	2.7%	16.3%	4.4%	23%	47.4%	4.8%	100%
	Skilled operational private sector workers	0.0%	2.1%	24.3%	18.0%	12.7%	4.6%	24.2%	10.5%	0.0%	2.8%	0.7%	0.0%	0.0%	0.0%	100%
	Professionals and technicians in the public and private sectors	1.1%	0.1%	6.3%	9.3%	7.9%	4.1%	22.9%	19.6%	2.0%	5.8%	5.4%	0.1%	14.6%	0.6%	100%
	Operational private sector workers in construction and manufacturing	1.3%	1.5%	15.1%	15.4%	10.7%	3.8%	21.7%	12.6%	2.6%	4.6%	3.8%	0.0%	7.0%	0.0%	100%
	Operational private sector workers in trade and basic services	0.0%	2.8%	29.8%	19.3%	12.2%	0.0%	20.8%	10.1%	1.2%	1.0%	0.0%	0.0%	1.5%	1.4%	100%
	Self-employed traders and vendors	0.9%	0.3%	7.2%	8.5%	6.8%	3.0%	28.4%	14.4%	4.5%	7.4%	7.4%	0.2%	9.9%	1.1%	100%
	Self-employed agricultural and construction workers	0.8%	1.3%	16.7%	16.4%	12.3%	2.6%	22.4%	12.1%	2.2%	6.1%	3.1%	0.0%	3.9%	0.1%	100%
	Domestic service workers	1.3%	1.2%	14.8%	10.3%	10.1%	2.5%	27.5%	10.4%	4.8%	7.2%	6.2%	0.0%	3.7%	0.0%	100%
	Total	0.9%	0.6%	8.9%	9.3%	7.3%	2.9%	21.4%	13.1%	2.9%	8.3%	5.0%	0.6%	17.5%	1.5%	100%

Source: Prepared by the authors using CASEN 2022 data.

**CHART 4:**
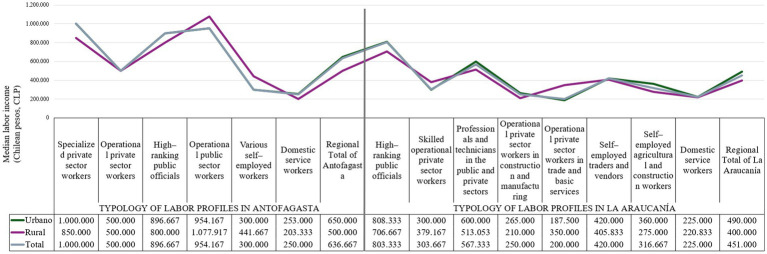
Median labor income (CLP) of the regional labor segments of Antofagasta and La Araucanía. Source: Prepared by the authors using CASEN 2022 data.

In La Araucanía, the High-ranking public group officials stands out for its high proportion of completed professional training (47.4%) and postgraduate training (4.8%), exceeding that observed in Antofagasta. In addition, the Professionals and technicians in the public and private sectors also have a high educational level, although with a lower concentration of completed university studies than the professional segment. On the other hand, the operating segments in commerce, construction, and manufacturing exhibit lower educational levels, with a high percentage of incomplete primary or secondary education (Chart 4).

In both regions, the relationship between academic qualifications and access to higher-skilled jobs is evident, although Antofagasta shows a more marked distribution toward secondary and technical training in the operational field. Araucanía shows a greater contrast between occupations with high levels of formal (professional) education and those with low levels of education, particularly in commerce and construction.

In both regions, most labor segments show a high proportion of workers with signed contracts, although with nuances (Table 13). In Antofagasta, the total labor force with a contract (90.1%) slightly exceeds that of La Araucanía (85.7%), although there are marked internal differences. For example, the specialized private sector workers in Antofagasta (99.3% with contract) and the High-ranking public Officials in La Araucanía (97.8%) stand out for their very high levels of formality. In contrast, in Antofagasta, other groups exhibit a high lack of contracts, with the obvious case of the various self-employed workers (94.7% without contract) and Domestic workers service workers (81.0%). In La Araucanía, on the other hand, an even more critical pattern is observed in some operational segments, such as the Operational private sector workers in construction and manufacturing (77.1% without contract) and Operational private sector workers in trade and basic services (98.8% without a contract). Unlike in Antofagasta, these latter segments of employment are not self-employment, but rather are subject to some type of dependency on an employer without a contractual legal intermediation.

**Table 13:** Distribution of employment segments according to signed employment contract, Antofagasta and La Araucanía.

Occupational profiles		In your main job, do you have a written employment contract?			
		Yes, signed	Yes, but not signed	No, it does not have one	Total
Typology of labor profiles in Antofagasta	Specialized private sector workers	99.3%	0.2%	0.5%	100%
	Operational private sector workers	91.3%	1.2%	7.5%	100%
	High-ranking public officials	85.9%	3.5%	10.6%	100%
	Operational public sector workers	67.9%	0.0%	32.1%	100%
	Various self-employed workers	3.2%	2.2%	94.7%	100%
	Domestic service workers	16.6%	2.4%	81.0%	100%
	Total	90.1%	0.8%	9.1%	100%
Typology of labor profiles in la Araucanía	High-ranking public officials	96.4%	1.0%	2.6%	100%
	Skilled operational private sector workers	63.8%	2.8%	33.4%	100%
	Professionals and technicians in the public and private sectors	97.8%	0.4%	1.7%	100%
	Operational private sector workers in construction and manufacturing	22.6%	0.4%	77.1%	100%
	Operational private sector workers in trade and basic services	1.2%	0.0%	98.8%	100%
	Self-employed traders and vendors	92.0%	0.6%	7.4%	100%
	Self-employed agricultural and construction workers	30.9%	0.0%	69.1%	100%
	Domestic service workers	20.1%	5.2%	74.7%	100%
	Total	85.7%	0.9%	13.4%	100%

Source: Prepared by the authors using CASEN 2022 data.

These contrasts demonstrate that, while Antofagasta maintains a higher level of formality in the private sector (driven by specialized and mining sectors), La Araucanía presents formalization gaps in low-skilled operational occupations in the private sector, in skilled occupations in the public sector, as well as in trade and service activities.

In both regions, self-employment and domestic work are associated with the absence of a signed contract. It is also important to highlight the significant proportion of absences in the Operational Public sector workers in Antofagasta. This reflects informality in these labor niches in both regions. Thus, the labor dynamics of both regions show similarities in the precariousness of the most vulnerable groups, but differ in the case of more skilled segments regarding the extent of formalization, which is more widespread in Antofagasta than in La Araucanía.

### 6.4 Intra-regional comparative synthesis

The evidence presented reveals patterns of relative specialization and functional concentration of employment between urban and rural areas, as well as distinct degrees of structural heterogeneity. The duality of labor segmentation establishes a separation between protected and unprotected groups in the labor market, which can be linked to marked spatial differentiation. Within this framework, the Antofagasta region presents a labor structure dominated by the specialized formal private sector, reflecting its mining and urban production profile, while La Araucanía shows greater labor heterogeneity, combining public employment, agricultural self-employment, and urban professionalization. Thus, La Araucanía reflects a more mixed labor market with a greater rural focus.

In Antofagasta, there is a strong urban specialization in formal private-sector wage employment—primarily in mining, industry, and services—while rural areas exhibit a dominant pattern of self-employment and informality. This configuration indicates a functionally fragmented structure, where public sector participation is marginal, reinforcing the urban–rural dualism and concentrating skilled employment in urban areas. In contrast, La Araucanía presents a more balanced urban–rural structure, with a less divided labor force. Although skilled employment is also concentrated in urban areas, the public sector has a significant presence in both territories, which contributes to mitigating territorial segmentation. Furthermore, rural self-employment shows greater diversification—including agriculture and construction—and operational work suggests the existence of links with local productive chains.

Beyond territorial differences, forms of labor segmentation are also observed associated with specific attributes of the workforce. The presence of foreign women in domestic work in Antofagasta, as well as self-employed agricultural and construction workers in rural areas of La Araucanía, demonstrates that factors such as nationality or employment type shape occupational segments excluded from the formal market. All of these groups operate under highly precarious and informal conditions, with limited opportunities for mobility. Recognizing these forms of segmentation allows for a more comprehensive approach, especially in contexts marked by migration dynamics and territorial inequalities.

## 7 Conclusion

In Antofagasta, six labor groups were identified, with specialized and operational employment in the private sector standing out, representing over 70% of the regional workforce. This configuration reflects an urban structure with high levels of formalization and labor protection, especially in specialized sectors. Informal employment is primarily concentrated among self-employed workers and domestic workers, creating a complementary informal economy linked to commerce and support services. Informal employment also exists among dependent and operational workers, both in the private and public sectors.

In contrast, Araucanía presents a typology with eight occupational groups that reflect a more heterogeneous labor structure. In this region, public officials, professionals, and technicians from both the public and private sectors represent nearly 45% of the employed population. The operational segments—mainly in commerce, construction, and manufacturing—and the self-employed maintain a strong connection with traditional activities, particularly agriculture. This configuration is associated with greater territorial dispersion of employment and a significant presence of rural labor, suggesting a less urbanized economy with a more diversified productive base.

These differences reveal structurally differentiated labor segmentation processes between the two regions. In Antofagasta, mining, urbanization, and sectoral concentration create a dual structure, in which rural areas are both very scarce and linked to informality. In La Araucanía, on the other hand, rurality and the expansion of the public sector outline a transition toward a more heterogeneous and territorially balanced employment mix.

As demonstrated by the factor analysis, at the interregional level, both regions present a similar structure around the formality/informality axis, which translates into a consistent distinction between dependent employment and self-employment. However, both regions diverge significantly in the second factorial dimension: while in Antofagasta it is associated with the predominance of labor protection, in La Araucanía the second factorial dimension reveals a feminized precarization of work, which would be due to the importance of domestic employment and female self-employment (both significantly precarious segments).

From an intraregional perspective, labor typologies also present significant differences. As already noted, Araucanía displays greater diversity in labor segments and a more marked territorial balance between urban and rural areas. This allows us to identify phenomena of differentiated functional specialization in each type of territory. In Araucanía, the rural labor market is not only denser but also more functionally diverse, including employment in state-run activities such as health, education, and local administration. Antofagasta, on the other hand, has a highly urbanized and concentrated structure, where public employment in rural areas is scarce and informality is mostly associated with these peripheral territories (Table 14).

**Table 14:** Descriptive summary of the labor segments of Antofagasta and La Araucanía.

Work segment		Main features
Typology of labor profiles in Antofagasta	Specialized private sector workers	They represent 44.1% of the regional working population. Employment is predominantly male (71%), with a significant presence in the mining sector (32.7%). The majority have a signed employment contract (99.3%) and complete professional education (30.2%). They are concentrated almost exclusively in urban areas (98.6%), and 7.7% are foreign nationals.
	Operational private sector workers	This represents 28.8% of the regional workforce. Fifty-four percent are men, and the majority work in commerce (25.5%) and construction. Ninety-one percent have signed contracts, with a predominance of secondary technical and humanities education. Ninety-six percent work in urban areas, and 22.6% are migrant.
	High-ranking public officials	They represent 3.9% of the regional total. The majority of the population is women (64%), working in education, health care, and public administration. 85.9% have signed contracts. They have a high level of professional education (43%).
	Operational public sector workers	They represent 1.9% of the regional workforce, predominantly men (72%), all of whom are in urban areas. There is a high concentration of public administration (98.2%), but only 67.9% have a signed contract.
	Various self-employed workers	This represents 19.2% of the region’s total working population. Comprised of 54% men, the workforce is highly active in commerce (34.5%) and manufacturing. A total of 94.7% of the population is unemployed. 91.8% work in urban areas, and 28.9% are foreign nationals.
	Domestic service workers	They represent 2% of the regional total. This segment is highly feminized (98%), concentrated in households as employers (94%), and has a high level of informality (81%).
Typology of labor profiles in la Araucanía	High-ranking public officials	They represent 22.1% of the region’s total employed population. The majority of these workers are women (60%), and they work in education, health care, and public administration. Ninety-six percent have signed contracts. They have a high level of professional education (47.4%).
	Skilled operational private sector workers	Comprising 1.5% of the regional workforce, this segment is 99% women. They work primarily in manufacturing and construction. 63.8% have signed contracts and complete secondary school.
	Professionals and technicians in the public and private sectors	This represents 22.4% of the regional total. Employment is predominantly male (96%) and urban (79.4%). Employment is concentrated in education, health care, and public administration. 97.8% have a signed contract, and 14.6% have a full professional degree.
	Operational private sector workers in construction and manufacturing	It accounts for 13% of the region’s employed population. Men predominate (57%). They are dependent workers with a high level of informality (77.1% without a contract). The majority of the population is in construction (39.4%), and 34.3% live in rural areas.
	Operational private sector workers in trade and basic services	They represent 1.6% of the total employed population in the region and are entirely women (100%). They are employed in retail trade (100%), with high informality (98.8% without a contract) and low educational levels. 82.5% live in urban areas.
	Self-employed traders and vendors	It accounts for 20.7% of the regional workforce. Women predominate (55%), with activities focused on retail trade. 80.2% live in urban areas.
	Self-employed agricultural and construction workers	Represents 13.4% of the regional total, with 92% being men. They work in agriculture (24.7%) and construction (31.9%), mostly without a contract (69.1%) and with low education. 41.5% work in rural areas.
	Domestic service workers	They account for 5.3% of the regional total. Segment highly feminized, although in minority as in Antofagasta (85% respectively), concentrated in homes as employers (90%), with high informality (74.7%).

Source: Prepared by the authors.

In both regions, skill gaps between men and women are closely linked to the feminization or masculinization of different labor segments. In Antofagasta, highly specialized private-sector workers are predominantly men and possess high educational attainment, whereas female-dominated segments, such as high-ranking public officials and domestic service workers, exhibit a higher concentration of primary or secondary education, reflecting more limited access to full professional qualifications. Similarly, in La Araucanía, senior public officials and skilled operational private-sector workers show a differentiated distribution by sex: men predominate in technical and agricultural occupations with lower educational levels, while women are concentrated in education, health, and domestic services, attaining higher education in senior positions but lower levels in informal or commercial occupations, highlighting a clear segmentation of job qualifications by sex and type of occupation.

These regional labor market trajectories reveal that, despite sharing a subordinate insertion in a peripheral-dependent economy like Chile’s, the productive and occupational structures of Antofagasta and La Araucanía differ substantially. Far from national homogeneity, labor segmentation reveals inter- and intraregional inequalities that respond to historical processes of productive specialization, urbanization, and differentiated public policies. Therefore, understanding the dynamics of contemporary capitalism in peripheral contexts requires incorporating regional analysis, recognizing structural heterogeneity and the different forms of territorial insertion in global value chains. This approach is key to designing employment, development, and well-being policies that effectively respond to local realities.

The results of this article invite the development of new research focused on the intersection of gender and ethnicity in peripheral contexts of the Global South. They also invite further investigation into the identification of qualitative conditions that can explain and provide greater evidence regarding the factors and relationships embedded in labor segmentation processes. This approach can lead to the generation of employment policy and program proposals that are more relevant in their targeting and in response to the characteristics of job demand and supply in regional labor markets. This article seeks to support new work that explores beyond what quantitative studies can offer and to foster ongoing reflection on the importance of the geography of labor markets and its opportunities for public policy.

## Data Availability

The original contributions presented in the study are included in the article/Supplementary material, further inquiries can be directed to the corresponding author.

## Footnotes

^1^For the contributions to the Inertia of the factor analysis of the two study regions, see Supplementary Tables 3, 4.

^2^Map 2 and Table 6 present the distribution of job profiles across urban and rural areas, calculated as the proportion of each segment within the total employed population of the respective territory. Map 2 provides this information at the provincial level (see footnote 5), whereas Table 6 presents the distribution at the regional level. Both sources allow for an analysis of the internal composition of each territory in terms of occupational profiles, offering a relative perspective on labor segmentation according to a defined territorial scale.

^3^These findings confirm the results of the factor analysis, where in La Araucanía the second dimension showed female job insecurity as a significant stratification factor in the region’s labor market.

^4^Which also implies the national indigenous population.

^5^These figures, calculated in Chilean pesos (CLP), have been converted to USD in the Supplementary Table 5.
