# Supplementary material for: Segmentation and regional analysis: labor typologies and geographic inequality in Antofagasta and La Araucanía, Chile
Source: Front Sociol. 2025 Oct 8;10:1636282. doi: 10.3389/fsoc.2025.1636282 (PMC12540500; doi:10.3389/fsoc.2025.1636282)
Supplement: Supplementary file 1 [file Supplementary_file_1.docx]

**Appendix**

*Table 11. Summary of the factorial model for the Antofagasta region*

| Dimensión | Alfa de Cronbach | Varianza contabilizada para | |
| --- | --- | --- | --- |
|  |  | Total (autovalor) | Inercia |
| 1 | 0,814 | 3,944 | 0,329 |
| 2 | 0,703 | 2,815 | 0,235 |
| Total |  | 6,759 | 0,563 |
| Media | ,768^a^ | 3,379 | 0,282 |

^a^ La media de alfa de Cronbach se basa en la media de autovalor.

Fuente: Elaboración propia con datos CASEN 2022.

*Tabla 12. Resumen del modelo factorial de la región de La Araucanía*

| Dimensión | Alfa de Cronbach | Varianza contabilizada para | |
| --- | --- | --- | --- |
|  |  | Total (autovalor) | Inercia |
| 1 | 0,815 | 3,955 | 0,33 |
| 2 | 0,667 | 2,576 | 0,215 |
| Total |  | 6,531 | 0,544 |
| Media | ,757^a^ | 3,265 | 0,272 |

^a^ La media de alfa de Cronbach se basa en la media de autovalor.

Fuente: Elaboración propia con datos CASEN 2022.

*Tabla 13. Medidas discriminantes de las variables en los ejes del ACM (Antofagasta)*

|  | 1 | 2 | Varianza Total |
| --- | --- | --- | --- |
| Sexo | 0,047 | 0,055 | 0,051 |
| En su trabajo o negocio principal, ¿trabaja como? | 0,720 | 0,179 | 0,45 |
| Tiene contrato de trabajo escrito | 0,243 | 0,114 | 0,178 |
| Tipo de contrato o acuerdo de trabajo | 0,133 | 0,012 | 0,073 |
| Afiliación a sistema previsional | 0,399 | 0,002 | 0,201 |
| Cotizó en sistema previsional | 0,326 | 0,786 | 0,556 |
| Actividad económica | 0,585 | 0,730 | 0,657 |
| CIUO 08 | 0,446 | 0,102 | 0,274 |
| Ingreso del trabajo corregido | 0,647 | 0,026 | 0,336 |
| Provincia | 0,021 | 0,008 | 0,015 |
| Pertenece a Etnia | 0,010 | 0,000 | 0,005 |
| Sistema previsional de salud | 0,367 | 0,801 | 0,584 |
| Totales (Resumen del Modelo) | 3,944 | 2,815 | 3,379 |

Fuente: Elaboración propia con datos CASEN 2022.

*Tabla 14. Medidas discriminantes de las variables en los ejes del ACM (La Araucanía)*

|  | 1 | 2 | Varianza Total |
| --- | --- | --- | --- |
| Sexo | 0,016 | 0,394 | 0,205 |
| En su trabajo o negocio principal, ¿trabaja como? | 0,722 | 0,605 | 0,664 |
| Tiene contrato de trabajo escrito | 0,264 | 0,182 | 0,223 |
| Tipo de contrato o acuerdo de trabajo | 0,209 | 0,025 | 0,117 |
| Afiliación a sistema previsional | 0,249 | 0,024 | 0,137 |
| Cotizó en sistema previsional | 0,476 | 0,015 | 0,246 |
| Actividad económica | 0,581 | 0,806 | 0,694 |
| CIUO 08 | 0,562 | 0,457 | 0,51 |
| Ingreso del trabajo corregido | 0,582 | 0,056 | 0,319 |
| Provincia | 0,007 | 0,002 | 0,004 |
| Pertenece a Etnia | 0,059 | 0,001 | 0,03 |
| Sistema previsional de salud | 0,226 | 0,007 | 0,117 |
| Totales (Resumen del Modelo) | 3,955 | 2,576 | 3,265 |

Fuente: Elaboración propia con datos CASEN 2022.
